# Influence of posteromedial corner injuries on clinical outcome and second-look arthroscopic findings after allograft transtibial anterior cruciate ligament reconstruction

**DOI:** 10.1186/s43019-020-00061-4

**Published:** 2020-08-10

**Authors:** Jun-Young Yoo, Hee-Gon Park, Soon-Min Kwon

**Affiliations:** Department of Orthopaedic Surgery, Dankook University College of Medicine, 119, Dandae-ro, Dongnam-gu, Cheonan-si, Chungnam 330-715 Republic of Korea

**Keywords:** Anterior cruciate ligament, Knee, Reconstruction, Posteromedial corner

## Abstract

**Background:**

The purpose of this study was to evaluate the influence of posterior medial corner (PMC) injuries on clinical outcome and second-look arthroscopic findings after anterior cruciate ligament (ACL) reconstruction.

**Methods:**

Seventy-eight consecutive patients underwent a second-look arthroscopic surgery after ACL reconstruction and magnetic resonance imaging (MRI) examination of the PMC. The patients were divided into a PMC intact group (*n* = 42) and a PMC injured group (*n* = 36). The stability and clinical outcomes were evaluated using the Lachman test, pivot-shift test, a KT-2000 arthrometer, and the Lysholm knee scoring scale. Graft tension and synovial coverage were evaluated in second-look arthroscopy.

**Results:**

The clinical function showed no significant differences regarding PMC injury. Although the graft tendon tension revealed no significant differences (*p* = 0.141), the second-look arthroscopic findings indicated that the PMC intact group showed better synovial coverage compared to the PMC injured group (*p* = 0.012).

**Conclusion:**

Patients who injured the PMC had poor synovial coverage as assessed by second-look arthroscopic findings after transtibial ACL reconstruction, even though clinical outcomes and stability showed no significant differences.

## Introduction

Recently, injury to the posteromedial corner (PMC) of the knee has been reported in several research works. Anatomically, the PMC is composed of five major components: the superficial medial collateral ligament (MCL), the deep MCL, the posterior oblique ligament (POL), the posterior horn of the medial meniscus, and the oblique popliteal ligament [1–4]. Medial-sided knee injuries are one of the most common knee ligament injuries encountered by orthopedic surgeons. These ligament injuries can occur in isolation or with concomitant meniscal or cruciate ligament injuries [1, 5]. The PMC functions as a primary stabilizer of the extended knee position: the load-bearing position of the knee in gait. In knee flexion, the PMC acts as a restraint to external rotation. Current biomechanical studies have revealed that the PMC contributes approximately one-third of the restraint to valgus stress in the extended knee [2, 6]. Furthermore, the posterior horn of the medial meniscus acts like a “brakestop,” providing anterior restraint in the absence of the anterior cruciate ligament (ACL) [7]. The ACL controls anterior movement of the tibia and inhibits extreme ranges of tibial rotation [8]. The ACL is also important in proprioceptive information and stabilization of muscular reflexes via a mechanoreceptor feedback system [9]. Therefore, combined ACL and PMC injuries are serious because the ACL and PMC are secondary stabilizers to each other.

PMC injuries are frequently associated with other ligament injuries such as those of the ACL, posterior cruciate ligament (PCL), or MCL. In the past several decades, PMC injuries have been managed in different ways including surgical or non-surgical treatment [7], and there is still controversy regarding their treatment. However, in recent years, numerous orthopedic surgeons have focused on surgical management for PMC-involved high-energy multiligamentous knee injuries [7, 10–13].

The purpose of the present study was to assess the influence of PMC injuries on clinical outcomes and second-look arthroscopic findings after ACL reconstruction. The hypothesis of this study was that, at least at the 2-year follow-up, PMC injuries are closely related with graft tension and clinical outcomes after ACL reconstruction.

## Materials and methods

After obtaining approval from our institutional review board, informed consent was obtained from 78 consecutive patients who underwent a second-look arthroscopic surgery after ACL reconstruction and magnetic resonance imaging (MRI) examination from January 2013 to November 2016.

The inclusion criteria were as follows: patients who underwent a second-look arthroscopic surgery after primary single-bundle ACL reconstruction using a modified transtibial technique with an allograft. Patients who had a history of previous surgery on the injured knee, multiligament injuries including the PCL, anterolateral ligament, or lateral collateral ligament, or arthritic changes were excluded. Patients who had an MRI scan more than 4 weeks after the initial trauma or in other hospitals were also excluded.

All surgeries were performed by a single, experienced orthopedic surgeon (HGP), using the modified transtibial technique. The tibial tunnel was made by drilling from the medial aspects of the proximal tibia to the femoral tunnel, using the modified method. The entering point of the tibial tunnel is located at the midpoint between the posterior cortex of the proximal tibia and the medial margin of the tibial tuberosity. A minimal notchplasty was performed to avoid complete removal of the remnant ACL at the tibial attachment site and prevent impingement of the grafted ACL. Since all surgeries were performed by a single surgeon, the remnant preservation was assumed to be the same. When there were meniscal or cartilage lesions, the proper procedure was performed. After forming the tibial tunnel, a transtibial femoral tunnel guide was inserted, and the rear angle of the guide was placed in the 10 o’clock position (right knee) knee or 2 o’clock position (left knee). Then, a tunnel with a depth of 30 mm and a diameter of 1 mm less than that of the graft was drilled to achieve approximately 1–2 mm of the femoral posterior wall. To fix the graft, the RigidFix technique (RigidFix; DePuy Mitek, Inc., Raynham, MA, USA) was used. The tibial tunnel was fixed again by using a post-tie after it was fixed with a bioabsorbable interference screw (Arthrex, Naples, FL, USA).

Joint flexion and extension were allowed starting from the day after surgery. Patients who did not undergo a meniscal repair procedure were allowed to perform partial weight-bearing activities for 2 weeks while wearing an ACL brace. After 2 weeks, full weight bearing was allowed. In patients who had a meniscal repair, partial weight bearing was performed for 6 weeks and the brace was worn for 6 weeks. We allowed jogging 3 months after surgery. Sports activity was allowed 6–9 months after surgery depending on the state of recovery.

**Fig. 1:**
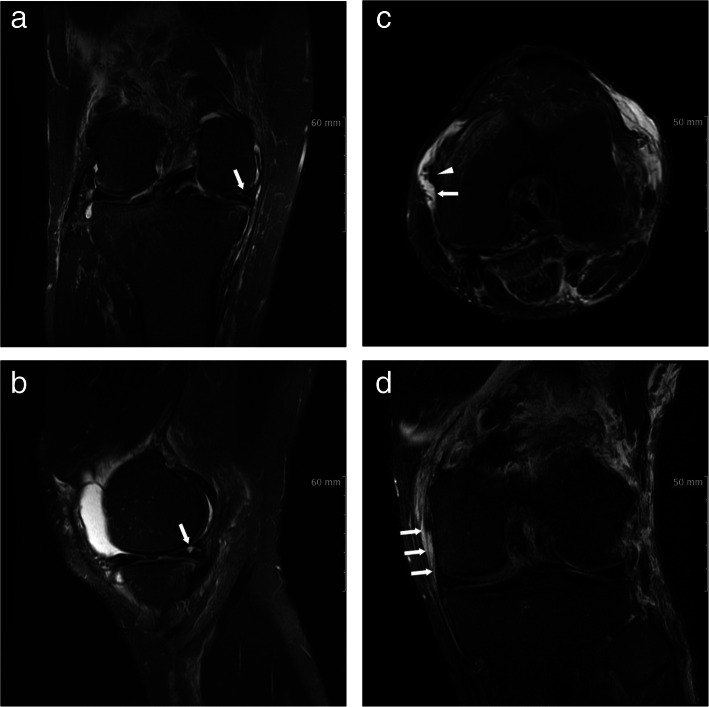
Magnetic resonance image of posteromedial corner injury. **a** Coronal fat-suppressed image of medial meniscus, showing complex tear of posterior horn (*arrow*). **b** Sagittal fat-suppressed image of medial meniscus, showing complex tear of posterior horn (*arrow*). **c** Axial fat-suppressed image of injury to the posterior oblique ligament (POL), showing edema around disrupted POL (*arrow*) and a normal femoral insertion of medial collateral ligament (*arrowhead*). **d** Coronal fat-suppressed image of injury to POL (*arrows*)

MRI evaluations were performed using 3.0 T devices (Philips Achieva, The Netherlands). Our MRI protocol included a coronal T1-weighted sequence; sagittal, axial, and coronal T2-weighted sequences with fat saturation; and a sagittal proton density-weighted sequence. The position of knee in full extension was recommended; however, if the patient was unable to fully extend the knee due to swelling or pain, a slightly flexed position was allowed. Examination results were assessed by two orthopedic surgeons (HGP) and (JYY). We considered a PMC as having a lesion if there was injury to at least one of the PMC structures. We considered lesions to be present if there were signal changes in the MRI or if injuries were found on arthroscopy, even partial injuries. We classified the PMC as normal or as having a lesion [6, 14–17] (Fig. 1).

**Fig. 2:**
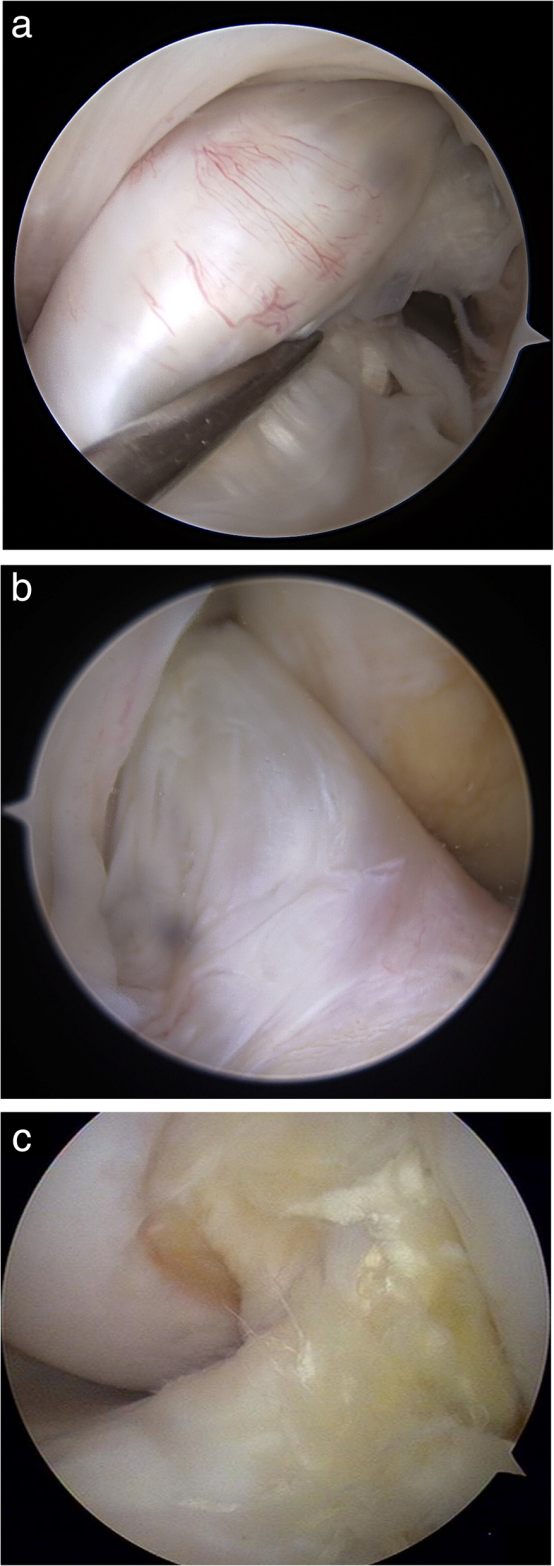
Classification of synovial coverage. **a** Good (nearly entirely covered); **b** half synovialization (> 50%); **c** pale (no coverage; < 50%)

**Fig. 3:**
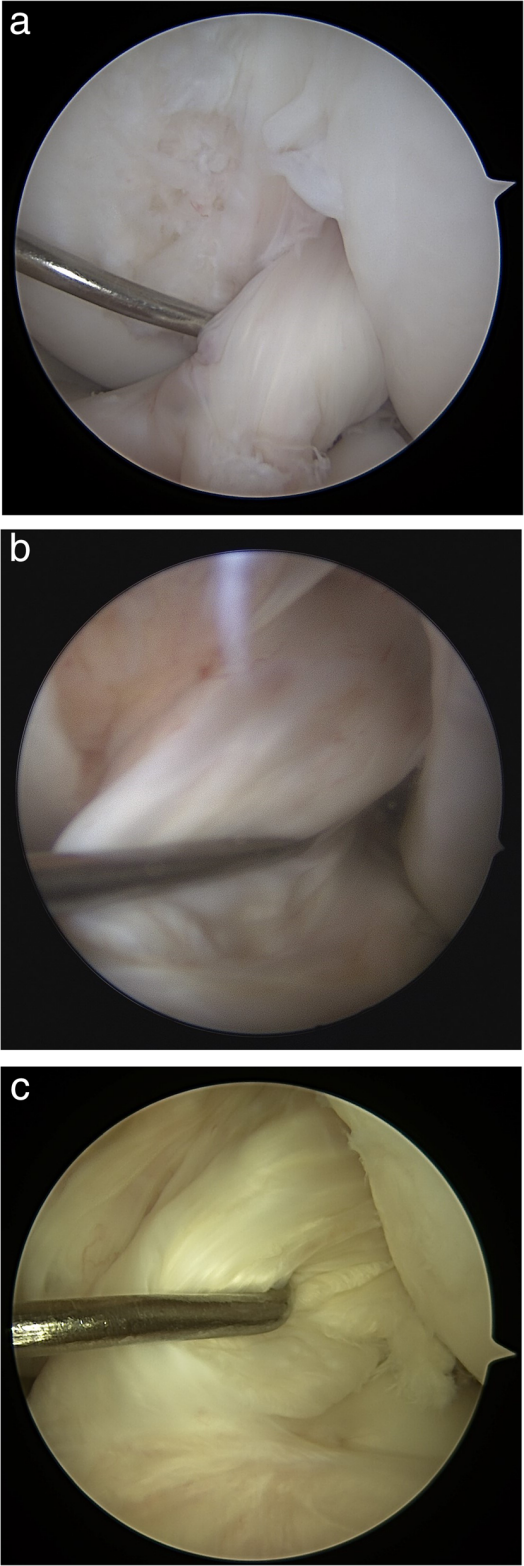
Classification of graft tension. **a** Normal (probing, < 2 mm); **b** lax (probing, > 2 mm but < 5 mm); **c** partial tear (probing, > 5 mm)

All the patients underwent a second-look arthroscopy and hardware removal at least 2 years after ACL reconstruction. All patients had given informed consent before surgery. The ACL graft status was evaluated by a single surgeon (HGP). Synovial coverage over the grafts was classified into the following three categories: good (nearly entirely covered), half (> 50%), and pale (no coverage; < 50%; see Fig. 2). Additionally, we used a modification of the classification system for ACL grafts described by Kim et al. to evaluate the tear of graft bundles during second-look arthroscopy [18]. In accordance with this system, the graft tear status was graded as normal (probing, < 2 mm), lax (probing, > 2 but < 5 mm), partial tear (probing, > 5 mm), and total tear (see Fig. 3). The hardware removal was performed after the ACL graft evaluation. Two weeks after surgery, running and sports were allowed.

All the patients were clinically evaluated before the initial and 2-year follow-up visits at second-look arthroscopic operation using a KT-2000 arthrometer, the Lachman test, pivot-shift test, and Lysholm knee scoring scale. The KT-2000 arthrometer test was performed at 30lbs to measure side-to-side difference in anterior translation with the knee positioned at 20° of flexion. In the pivot-shift test, the knee was graded as normal, close to normal, and abnormal. The Lachman test and pivot-shift test were perfomed by a single surgeon (HGP). The Lysholm knee scoring scale was used for general evaluation of the knee [19].

To evaluate the normal distribution of the continuous data, the Kolmogorov-Smirnov test was performed, the continuous variable was analyzed using an independent *t* test, and the non-continuous variable was analyzed using the Pearson chi-square test. All statistical analyses were performed using the Statistical Package for Social Sciences version 20.0 (SPSS, Inc., an IBM Co., Chicago, IL, USA). Statistical significance was considered at a *p* value of < 0.05 for all the analyses.

## Results

Of the 78 patients included in this study, there were 42 patients with intact PMCs and 36 patients with injured PMCs. There was no significant difference between the two groups with respect to age, sex, body mass index, mean duration to follow-up second-look arthroscopy, location of meniscal tears, or surgical treatment for the meniscus (see Table 1).

**Table 1:** Demographic data

	PMC intact group (*n* = 42)	PMC injured group (*n* = 36)	*P*
Age	31.9	29.2	n.s.
Sex (male:female)	19:2	7:2	n.s.
Body mass index	25.0	24.2	n.s.
Mean follow-up to second-look arthroscopy (months)	27.4	25.9	n.s.
Medial meniscus tear	2^a^	24	n.s.
Lateral meniscus tear	10	12	n.s.
Medial and lateral meniscus tear	0	2	n.s.
Meniscectomy	4	12	n.s.
Meniscus repair	2	10	n.s.

^a^These two had tears of the anterior horn, not tears of the posterior horn of the medial meniscus

All patients undergoing meniscus repair were well healed without re-tear, and ACL grafts in all patients had no partial or total tears.

Of the 42 patients in the PMC intact group, 24 (57.1%) showed a good synovial coverage, 18 (42.9%) showed half synovial coverage, and none showed pale synovial coverage. Of the 36 patients in the PMC injured group, 30 (83.3%) showed a good synovial coverage, 6 (16.7%) showed half synovial coverage, and none showed pale synovial coverage. Synovial coverage in association with PMC injuries showed a statistically significant difference between the two groups (see Table 2). The PMC injured group showed poor synovial coverage.

**Table 2:** Arthroscopic findings following PMC injury

	PMC intact group (*n* = 42)	PMC injured group (*n* = 36)	*P*
Tension			0.141
Normal	36 (85.7%)	26 (72.2%)	
Lax	6 (14.3%)	10 (27.8%)	
Partial tear	0	0	
Complete tear	0	0	
Synovial coverage			0.012
Good	24 (57.1%)	30 (83.3%)	
Half	18 (42.9%)	6 (16.7%)	
Pale	0	0	

Of the 42 patients in the PMC intact group, 36 (85.7%) showed a normal graft, 6 (14.3%) showed a lax graft, and none had partial or total tears. Of the 36 patients in the PMC injured group, 26 (72.2%) showed a normal graft, 10 (27.8%) showed a lax graft, and none had partial or total tears. Graft tension showed no statistically significant difference between the two groups (Table 2).

The mean postoperative Lysholm scores were, respectively, 92.8 and 93.9 for the PMC intact group and the PMC injured group. The mean KT-2000 arthrometer values were similar at both pre-operation and at the 2-year follow-up, but no statistical difference was found between the groups. Furthermore, the Lachman test and pivot-shift test showed statistically comparable results. However, there was no statistically significant difference in clinical outcomes (see Table 3). Also, the correlation between the Lachman test and graft tension was not significant.

**Table 3:** Clinical outcomes comparison

	PMC intact group (*n* = 42)	PMC injured group (*n* = 36)	*P*
Mean last Lysholm score	92.8	93.9	n.s
Mean KT-2000			
Pre-operation	4.9	4.7	n.s
Last follow-up	1.5	1.2	n.s
Lachman test			
Pre-operation			n.s
Normal	4	6	
Close to normal	2	2	
Abnormal	36	28	
Last follow-up			n.s
Normal	32	34	
Close to normal	10	2	
Abnormal	0	0	
Pivot-shift test			
Pre-operation			n.s
Normal	4	6	
Close to normal	8	6	
Abnormal	30	24	
Last follow-up			n.s
Normal	42	36	
Close to normal	0	0	
Abnormal	0	0	

Of the 78 patients, there were 20 patients with an injured MCL, 9 with POL injuries, 24 patients with an injured posterior horn of the medial meniscus, and 6 with oblique popliteal ligament injuries (see Table 4).

**Table 4:** Distribution of the injured structure of PMC

Injured structure	
Medial collateral ligament	20
Posterior oblique ligament	9
Posterior horn of medial meniscus	24
Oblique popliteal ligament	6

## Discussion

The MCL provides the primary valgus restraint in the flexed knee and is an external rotation stabilizer [20, 21]. With extension, the PMC becomes the primary stabilizer to valgus stress and prevents posterior tibial translation [2, 3, 6, 7]. Consequently, PMC injuries combined with ACL injuries are significantly different and more serious than isolated ACL rupture and make the knee unstable in valgus motion and rotations. To the best of our knowledge, this study is the first to evaluate the influence of PMC injuries on second-look arthroscopic findings after ACL reconstruction.

In this study, there was poor synovial coverage at the second-look arthroscopic examination in the PMC injured group. Synovialization is an important factor in graft healing and survival of the graft, and thus laxity. It is necessary to restore proprioception, because most of the mechanoreceptors of the ACL are found in the subsynovial layer [9, 22]. Thus, preservation of the synovium and promotion of synovialization of the graft after ACL reconstruction are considered to be important in restoring proprioceptive function. According to a study by Katayama et al. [23], stability was associated with proprioceptive function. The authors thought that PMC injuries could decelerate synovial coverage of the reconstructed ACL because PMC injuries usually lead to valgus instability.

However, clinical outcomes showed no significant difference between both groups. These results showed that clinical outcomes did not entirely reflect graft state in second-look arthroscopic examinations, because clinical outcome is the result of a combination of various conditions, although synovial coverage is one of the most important factors.

Bollen et al. [24] reported 183 cases of ACL reconstructed knee, of which 9.3% were PMC injured. Our study had 36 cases (46%) that were PMC injured. Pandey et al. [7] reported 35 patients with MCL-PMC injury, of whom 20 patients had ACL injuries. They reported that primary MCL-PMC repair renders the knee stable and provides a superior clinical outcome. In our study, ACL was reconstructed but PMC was conserved.

There are several limitations to the present study. First, this study is a retrospective, not a case-controlled, study. Second, we also presumed that preoperative clinical stability would be influenced by PMC injury, but the results showed no difference—not only postoperatively in the ACL reconstructed knee but also preoperatively in the ACL deficient knee. This can be demonstrated by the selection bias: in this study ACL reconstruction was performed in the cases with persistent instability, and stable ACL deficient knees without PMC injury were excluded. Third, the influence of PMC injuries on ACL reconstruction failure could not be analyzed. In the present study, none of the cases had total rupture of the ACL graft because all re-ruptured cases were excluded in line with the exclusion criteria. Fourth, proprioception testing according to synovialization was not conducted. Only synovialization was assessed by arthroscopy. Further studies to evaluate the relationship between the extent of synovialization and proprioception may be needed. Fifth, the extent of remnant ACL preservation may affect synovial coverage, but this was not considered. Since all surgeries were performed by a single surgeon, the remnant preservation was assumed to be the same. Sixth, the degree of meniscectomy and valgus instability at final follow-up in an MCL injured patient can affect ACL graft healing, but this was not considered. Further studies to evaluate the relationship between degree of meniscectomy and valgus instability may be needed.

## Conclusion

Patients who injured the PMC had poor synovial coverage in the second-look arthroscopic examination after transtibial ACL reconstruction. However, there were no significant differences in clinical outcomes and stability.

## Data Availability

The data and materials that support the findings of this study are openly available.

## Abbreviations

ACL: Anterior cruciate ligament
ALL: Anterior lateral ligament
MCL: Medial collateral ligament
MRI: Magnetic resonance imaging
PMC: Posteromedial corner
